# Impact of ultrasonication, ozonation, and their combination on the preservation of novel clean-label functional drink of strawberry-cantaloupe incorporated with *Spirulina platensis* and orange peel extracts

**DOI:** 10.1016/j.ultsonch.2025.107455

**Published:** 2025-07-05

**Authors:** Kashmala Chaudhary, Samran Khalid, Najla AlMasoud, Taghrid S. Alomar, Sadia Ansar, Ahmed Fathy Ghazal, Abderrahmane Aït-Kaddour, Rana Muhammad Aadil

**Affiliations:** a National Institute of Food Science and Technology, University of Agriculture, Faisalabad 38000, Pakistan; b Department of Chemistry, College of Science, Princess Nourah bint Abdulrahman University, PO Box 84428, Riyadh 11671, Saudi Arabia; c Agricultural Engineering Department, Faculty of Agriculture, Suez Canal University, Ismailia 41522, Egypt; d Université Clermont Auvergne, INRAE, VetAgro Sup, UMRF, 63370 Lempdes, France; e Department of Food Technology, Faculty of Agroindustrial Technology, University of Padjadjaran, Sumedang 45363 Jawa Barat, Indonesia

**Keywords:** Functional drink, Thermal treatment, Non-thermal preservation, Ultrasonication, Ozonation, Combined treatment, Shelf life

## Abstract

Consumer demand for sustainable, nutrient-rich, and clean-label beverages is rapidly increasing, which is driving the need for innovative preservation techniques for such sensitive beverages in place of conventional chemical preservatives and thermal treatments, which have several drawbacks. These advanced methods need to be effective in maintaining quality, retaining bioactivity, ensuring safety, and extending the shelf life of these novel sensitive beverages. This study focused on the sustainable non-thermal preservation of a novel clean-label functional drink formulated from strawberry and cantaloupe juice, incorporated with *Spirulina platensis* and orange peel extracts. The drink was subjected to conventional pasteurization (90 °C for 1 min), ultrasound treatment (300 W power, 25 kHz frequency, 10 min at 25 °C), ozone treatment (30 mg/L concentration, 1 L/min flow rate, 10 min at 25 °C), and a combined ultrasound and ozone treatment, applying the same parameters for 5 min each. The treated drink samples were stored at 4 °C for two months, and various analyses were conducted over a fifteen-day period to assess preservation outcomes. The best results, in terms of maintaining quality, retaining bioactivity, ensuring safety, and enhancing shelf life, were achieved with the combined treatment of ultrasonication and ozonation. This research highlights the importance of synergistic non-thermal technologies over single treatments in the preservation of beverages. Additionally, the presence of antimicrobial and antioxidant compounds from the algal and plant extracts in the drink formulation also contributed to its extended shelf life. These findings demonstrate the potential of sustainable and environmental friendly non-thermal technologies for preserving functional beverages without compromising their functional attributes. The findings pave the way for future research and industrial-scale adoption of sustainable preservation methods, contributing to a healthier and more sustainable food system.

## Highlights

• A clean-label functional drink was formulated using strawberry and cantaloupe juice, with the addition of extracts.
• The effectiveness of different methods for preserving the functional drink was assessed.
• The synergistic effect of ultrasonication and ozonation offered superior preservation of this drink.
• These functional drinks treated with non-thermal techniques could sustain a shelf life of 2-4 months.

## Graphical abstract

**Figure:**
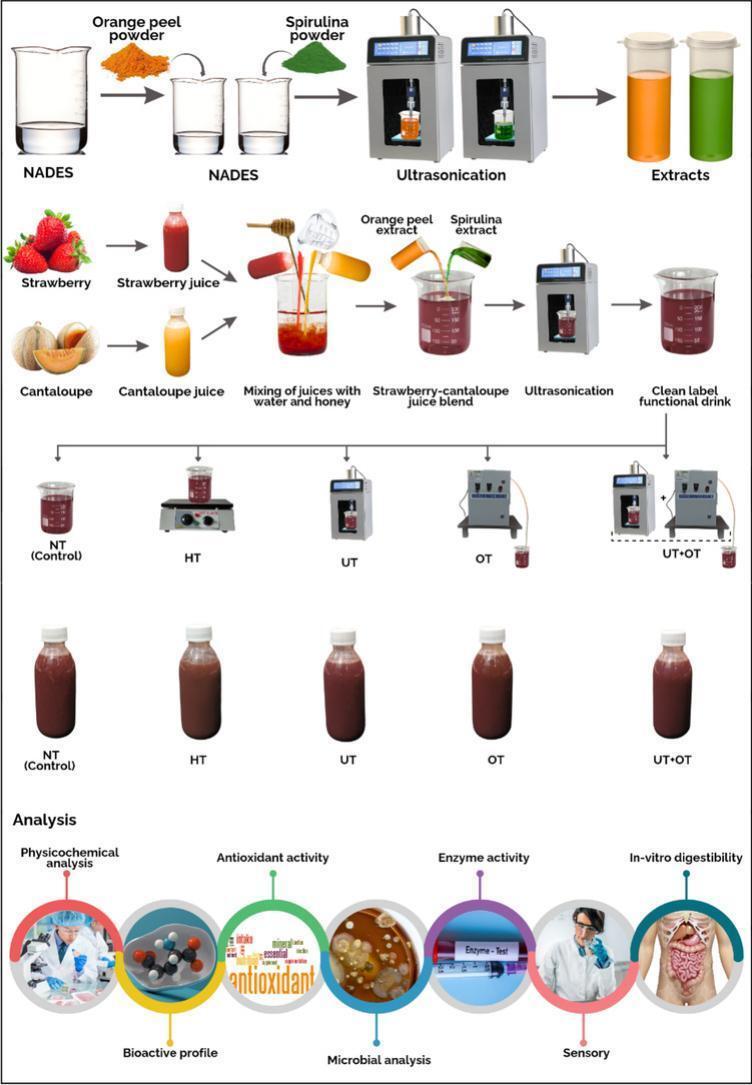

## 1 Introduction

Growing consumer awareness of health, nutrition, and sustainability has amplified the demand for sustainable, healthy, nutrient-rich, clean-label functional beverages [1,2]. Functional beverages provide health benefits beyond basic nutrition, often by reducing disease risk or improving overall well-being [3,4]. Clean-label beverages are developed with simple, natural, and recognizable ingredients, free from artificial additives, synthetic chemicals, and overly processed components [2,5]. Functional beverages are a fast-growing segment of the functional food industry, formulated with bioactive compounds that offer health benefits beyond hydration [6]. They often contain probiotics, prebiotics, vitamins, minerals, antioxidants, herbal extracts, amino acids, and plant-based compounds with health-promoting properties [1,7].

The effective preservation is the most crucial aspect for the functional beverage industry, as it ensures quality maintenance, retains bioactivity, guarantees long-term safety, and extends the shelf life of these novel, sensitive beverages. Common preservatives used in beverages, such as potassium sorbate, sodium benzoate, sulfur dioxide, sorbic acid, calcium disodium, and citric acid, help prevent spoilage, inhibit microbial growth, and extend shelf life. However, despite their effectiveness, these chemical preservatives come with several drawbacks, including off-flavors, potential allergic reactions, and various health concerns [8]. Traditional thermal treatments, such as pasteurization, have long been used for beverage preservation, but they pose several drawbacks, including the degradation of heat-sensitive nutrients and bioactive compounds of functional beverages [9]. Many studies have been conducted on the use of natural extracts as preservatives in beverages, demonstrating exceptional results in their ability to enhance preservation based on their antimicrobial, antioxidant, and shelf-life-extending properties [2,10,11]. However, effective preservation methods are still required that can ensure long-term preservation. To address these challenges, advancements in food preservation have led to the development of innovative non-thermal technologies, which could be very effective in preservation of functional beverages [5,12]. Techniques such as ultrasonication (US), high-pressure processing, pulsed electric field, pulsed light, ozonation (OZ), cold plasma, and irradiation have shown great potential in maintaining the integrity of bioactive ingredients of sensitive food products [4]. These non-thermal methods provide a promising approach for the sustainable preservation of functional beverages, aligning with consumer demand and sustainability principles. Among these, the most effective methods are US, a processing technique that utilizes high-frequency sound waves (typically between 20 kHz and 100 kHz) to create cavitation effects, and OZ, which involves the use of ozone (O_3_), a powerful oxidizing agent, for food preservation [8].

Many studies have evaluated the impact of non-thermal technologies on beverage preservation, with some specifically exploring their effectiveness in functional drinks also. However, this research investigated the synergistic effect of US and OZ on preserving a novel clean-label functional drink made from a strawberry and cantaloupe juice blend, added with *S. platensis* and orange peel extracts, without any additives or preservatives. Comprehensive analyses were conducted to assess the drink’s nutritional composition, bioactive compounds, in-vitro digestion, antimicrobial properties, and sensory attributes. This study is unique in its development of a sustainable, clean-label beverage and its application of a combined non-thermal preservation approach. The ultimate goal is to showcase the potential of sustainable and eco-friendly non-thermal methods for preserving clean-label functional beverages, with the aim of scaling up to an industrial level and revolutionizing the food industry.

## 2 Materials and methodology

### 2.1 Raw materials

Fully mature, fresh, and ripe strawberries (*Fragaria × ananassa*, cultivar: Chandler) were procured from farms located in Kasur, Pakistan, at commercial maturity (fully red color, firm texture, and sugar-to-acid ratio (8.5) optimal for harvest). Fully ripe cantaloupes (*Cucumis melo*, cultivar: Hales Best) were sourced from farms in Bahawalpur, Pakistan, at physiological maturity (uniform netting, yellowish skin color, and characteristic aroma indicating ripeness). All fruits selected were intact, free from diseases, and exhibited uniform characteristics in terms of color, size, and ripeness. Orange peel powder and *S. platensis* powder were purchased online via Daraz.pk, and honey was acquired from a local village near District Faisalabad, Pakistan. All chemicals utilized in this study were of laboratory grade and obtained from Sigma-Aldrich (Merck KGaA, Darmstadt, Germany).

### 2.2 Preparation of NADES and extracts

Natural deep eutectic solvent (NADES) utilized for ultrasound assisted extraction (UAE) was prepared from lactic acid, serving as the hydrogen bond donor, and choline chloride, acting as the hydrogen bond acceptor. The components were mixed in a 2:1 M ratio and heated to 80 °C with continuous stirring until a clear, homogeneous solution formed. To adjust the viscosity, 30 % water was added to the eutectic mixture. The UAE with NADES was conducted based on the approach detailed in our previous related study [2]. Extraction suspensions were prepared by individually mixing *S. platensis* and orange peel powder into the NADES. The suspensions, with a sample-to-solvent ratio of 1:30 g/mL, were pre-soaked for 30 min to promote cell disruption. UAE was performed using a probe-type ultrasonicator (SCIENTZ-750F, Ningbo, China) operating at a pulsating mode (10 s on: 5 s off) with a power of 300 W and a frequency of 25 kHz for 30 min. The temperature was maintained at approximately 40 °C by placing the flask in an ice bath to mitigate heat accumulation. After the processes, the suspensions were centrifuged at 10,000 rpm for 10 min using a refrigerated benchtop centrifuge (416KS, Sigma, Osterode, Germany), and the supernatants were collected for analysis and further use as a clean label ingredients. Protein content was measured using the Kjeldahl method, while lipid content was evaluated through Soxhlet extraction. The total phenolic content (TPC) was determined using the Folin-Ciocalteu assay, and the total flavonoid content (TFC) was analyzed via the aluminum chloride method. Antioxidant activity was tested using DPPH free radical scavenging, whereas chlorophyll *a*, chlorophyll *b*, and carotenoids were quantified through spectrophotometry. Additionally, carotenoids were precisely quantified using high-performance liquid chromatography (HPLC).

### 2.3 Development of functional drink

**Fig. 1:**
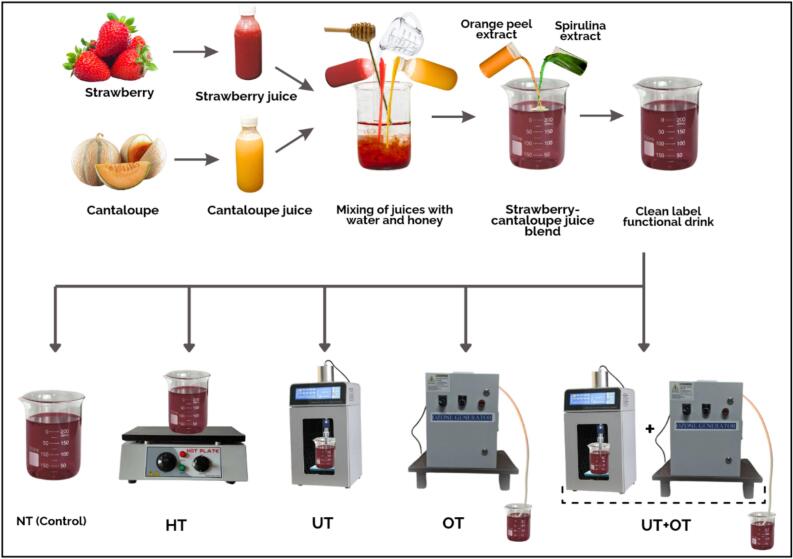
Development of clean-label functional drink and its preservation using different different methods (NT, HT, UT, OT, UOT).

The detailed methodology for developing various formulations of a functional drink is outlined in our previous study [2]. The optimal formulation, determined through comprehensive analysis, was selected for preservation based on the findings of the prior research. A concise overview of that drink formulation development process is as follows, the fruits were sorted, washed, peeled, and pulped, after which the pulp was filtered to obtain juice. A ratio of 35 % strawberry and 35 % cantaloupe juice was used, with 5 % honey added as a sweetener and 0.5 % pectin incorporated as a stabilizer. Additionally, 15 % water was added, followed by 5.0 % *S. platensis* and 5.0 % orange peel extracts, to achieve a final volume of 100 mL as shown in Fig. 1.

### 2.4 Preservation of functional drink

This natural functional drink, free from preservatives and potentially harmful additives, was subjected to non-thermal treatments for preservation. Preliminary trials involving non-thermal treatments with varying durations and power levels were conducted to identify the parameters that yielded the best outcomes, which were then adopted for this study. A total of four treatments were applied to the functional drink and NT, served as the control, with no preservation technique applied. For HT, conventional pasteurization was carried out at 90 °C for 1 min. In UT, US was performed, operating at a pulsating mode (10 s on: 5 s off) with 300 W power and a frequency of 25 kHz for 10 min while maintaining the temperature at 25 °C. The OT treatment involved OZ, using an ozone generator (Ozonia® TOGC, Saint-Maurice, Switzerland) at an ozone concentration of 30 mg/L with a flow rate of 1 L/min for 10 min at room temperature (25 °C). Finally, UOT combined US and OZ, with each process applied for 5 min under the previously mentioned conditions (Fig. 1). The treated drinks were subsequently stored at 4 °C in sterilized plastic bottles for two months. All analyses, except for in-vitro digestibility and sensory evaluation (conducted only on the first day), were performed at 15-day intervals to assess the quality, bioactivity, safety, and shelf life of the functional drink throughout the storage period.

### 2.5 Drink analysis

#### 2.5.1 In-vitro digestibility

In-vitro digestibility of the samples was assessed right after preservation treatments by using the method followed by Fradinho et al. [13] using a simulated gastrointestinal digestion process. Briefly, 1 mL of each sample formulation was placed into a 250 mL conical flask. To this, 25 mL of phosphate buffer (0.1 M, pH 6.0) was added and mixed thoroughly. The pH was then adjusted to 2.0 by adding 10 mL of 0.2 M HCl. Subsequently, 3 mL of freshly prepared pepsin solution, containing 30 mg of porcine pepsin with an activity of 0.8 FIP units/mg, was introduced. The mixture was incubated at 39 °C with constant agitation for 3 h to simulate gastric digestion. After this phase, the pH was raised to 6.8 by adding 10 mL of phosphate buffer and 5 mL of 0.6 M NaOH. Next, 10 mL of pancreatin solution (prepared in 50:50 ethanol:water) containing 500 mg of porcine pancreatin (42,362 FIP units/g) was added, and the samples were incubated further at 39 °C for 9 h under continuous agitation to simulate intestinal digestion. A reagent blank without sample was run simultaneously as a control. Post-digestion, undigested residues were collected by centrifugation, washed twice with deionized water, and filtered through glass fiber membranes. Both residues and membranes were dried initially at 60 °C for 6 h, followed by drying at 55 °C until a constant weight was obtained. The in-vitro digestibility (%) was calculated using the formula present in Eq. (1).

(1) $$Digestibility\;\left(\%\right)=\;\left[\left(Wi-Wu\right)/Wi\right]\times 100$$

*Wi* = Initial weight

*Wu* = Undigested weight.

#### 2.5.2 Sensory evaluation

Sensory evaluation of all preserved drink samples was conducted using a structured 9-point hedonic scale, where 1 denoted “extremely dislike” and 9 indicated “extremely like.” The assessment was performed after preservation treatments to evaluate the sensory impact of different formulations. A semi-trained panel of fifteen judges (aged 30–50 years) was recruited based on their prior experience in sensory analysis, frequent participation in food evaluation panels, and successful completion of standard sensory training sessions. Each judge provided informed consent prior to participation, with the right to withdraw at any time without penalty. Sensory analysis was carried out in individual booths under controlled conditions of ambient temperature (22 ± 2 °C), neutral lighting, and minimal distractions to ensure objective responses.

Approximately 20 mL of each drink sample was served in coded, odorless, transparent plastic cups at 10 ± 1 °C. Judges were instructed to evaluate five key sensory attributes, appearance, aroma, flavor, mouthfeel, and overall acceptability. Palate cleansing between samples was ensured using room-temperature distilled water and unsalted plain crackers. A 2-min interval was observed between successive samples to prevent sensory fatigue. Scores for each attribute were recorded and averaged across all judges. The results were expressed as mean sensory scores ± standard deviation.

#### 2.5.3 pH, titratable acidity (TA), and total soluble solids (TSS)

The pH of samples was measured using a calibrated digital pH meter, standardized with buffer solutions at pH 4.0 and pH 7.0 before each set of measurements to ensure accuracy. Approximately 25 mL of each sample was used for each pH determination, performed at room temperature (22 ± 2 °C). TA was determined using acid–base titration. A 10 mL aliquot of sample was diluted with 50 mL of distilled water, and the mixture was titrated against 0.1 N NaOH using 1 % phenolphthalein solution as an indicator. The endpoint was marked by the appearance of a faint, stable pink color. The volume of NaOH used was recorded and TA was calculated as % citric acid equivalent using the formula given in Eq. (2). TSS content was assessed using a digital refractometer at 20 °C, and results were expressed as °Brix. Prior to measurement, the refractometer was calibrated with distilled water. About 2–3 drops (∼0.5 mL) of each sample were placed on the prism surface for each reading, and three independent measurements were taken for each sample.

(2) $$\mathrm{TA}\;\left(\%\;citric\;acid\right)=\;\left(\;V\;\backslash times\;N\;\backslash times\;0.064\;\backslash times\;100\right)\;/\;S$$

*V* = volume of NaOH used (mL)

*N* = normality of NaOH

*0.064* = equivalent weight of citric acid in g/meq

*S* = volume of sample (mL).

#### 2.5.4 Total phenolic content (TPC) and total flavonoid content (TFC)

TPC and TFC of the samples were determined by the procedure used by Ebrahimi & Rastegar. [14]. To prepare the methanolic extract, 10 mL of the drink sample was mixed with 10 mL of 80 % (v/v) methanol solution, resulting in a 1:1 (v/v) ratio. The mixture was homogenized thoroughly and then centrifuged at 5,000 rpm for 10 min at 4 °C. The clear supernatant obtained was used as the sample extract for subsequent analyses.

For TPC determination, a reaction mixture was prepared by mixing 0.5 mL of the methanolic extract with 1.5 mL of 5 % (w/v) sodium carbonate solution and 1.5 mL of Folin-Ciocalteu reagent diluted 1:10 (v/v) with distilled water. The mixture was incubated at room temperature (approximately 25 °C) for 90 min. After incubation, the absorbance was measured at 750 nm using a UV–Visible spectrophotometer. Gallic acid was used to prepare the calibration curve, and TPC results were expressed as mg gallic acid equivalents (GAE)/100 mL of drink sample. For TFC determination, 0.5 mL of the methanolic extract was mixed with 0.1 mL of 10 % (w/v) aluminum chloride (AlCl_3_) solution and 0.1 mL of 1 mmol/L potassium acetate solution. The mixture was incubated at room temperature for 30 min before measuring absorbance at 415 nm. Quercetin was used as the standard to generate a calibration curve, and TFC results were expressed as mg quercetin equivalents (QE)/100 mL.

#### 2.5.5 Total carotene content (TCC)

The UV-spectrophotometer (Shimadzu UV-2600, Shimadzu Corporation, Japan) was used to assess the TCC by the method of Abidoye et al. [15]. A solvent mixture comprising hexane, acetonitrile, and ethanol in the ratio of 50:25:25 (v/v/v) was used for extraction. Specifically, 1 mL of the sample was mixed with 50 mL of the solvent mixture (1:50, v/v) in a centrifuge tube. The mixture was stirred using a magnetic stirrer for 15 min to promote effective extraction of carotenoids. Then, 5 mL of distilled water was added to the mixture to assist with phase separation. The solution was homogenized for 5 min and then left undisturbed for another 5 min to allow the upper hexane layer to separate. The upper hexane layer containing the extracted carotenoids was carefully collected, and its absorbance was measured at 450 nm using hexane as a blank and the values of TCC were expressed in mg/100 mL.

#### 2.5.6 Antioxidant activity

The antioxidant activity of the samples was measured by conducting three assays using the methods of Agunbiade et al. [16], Bendaali et al. [17], and Ashfaq et al. [18]. The DPPH (2,2-diphenyl-1-picrylhydrazyl) free radical scavenging activity was measured following standard procedures. A reaction mixture was prepared by combining 400 µL of methanolic extract (prepared from a 1:1 v/v extraction of a 10 mL sample and 80 % methanol), 200 µL of distilled water, and 600 µL of 0.1 mM DPPH solution in methanol. The mixture (total volume: 1.2 mL) was incubated in the dark at room temperature (22 ± 2 °C) for 30 min. The absorbance was measured at 517 nm using a UV–Vis spectrophotometer. The DPPH radical scavenging activity was calculated using the Eq. (3) and expressed as % inhibition. Since the extract was prepared from a known volume of juice, the results represent antioxidant activity per 100 mL of original drink volume.

The ABTS•^+^ working solution was prepared by reacting 7 mM ABTS stock solution with 2.5 mM potassium persulfate (1:1, v/v), and the mixture was incubated in the dark at room temperature for 16 h to allow radical formation. Before use, the solution was diluted with ethanol to an absorbance of 0.70 ± 0.02 at 734 nm. For the assay, 30 µL of the sample extract was mixed with 3 mL of ABTS•^+^ solution and incubated in the dark for 6 min. The absorbance was then measured at 734 nm. Results were quantified based on a Trolox standard curve and expressed as µmol TE/mL. The FRAP reagent was freshly prepared by mixing 25 mL of 300 mM acetate buffer (pH 3.6), 2.5 mL of 10 mM TPTZ (2,4,6-tripyridyl-s-triazine) solution in 40 mM HCl, and 2.5 mL of 20 mM FeCl_3_·6H_2_O solution. For the reaction, 200 µL of the sample extract was added to 1.8 mL of FRAP reagent, and the mixture was incubated at 37 °C in the dark for 10 min. The absorbance was recorded at 593 nm. Antioxidant capacity was calculated using a standard curve prepared with FeSO_4_·7H_2_O and expressed as mol Fe^2+^ equivalents/mL.

(3) $$\mathrm{DPPH}\;\left(\%\right)\;inhibition=\left(A0-As\right)/A0\;\times 100$$

*A_0_* = absorbance of the control (DPPH solution without sample)

*A_s_* = absorbance of the sample.

#### 2.5.7 Ascorbic acid

The ascorbic acid content was determined using the indophenol titration method, as described in the study Chettri et al. [19]. A 5 mL sample was combined with 5 mL of 0.5 % oxalic acid solution to prevent oxidation and then filtered through Whatman No. 1 filter paper. The resulting filtrate was titrated with freshly prepared 2,6-dichlorophenolindophenol (DCPIP) solution until a stable pink endpoint was achieved.

#### 2.5.8 Color

The color analysis of the functional drink was conducted using a portable colorimeter (Konica Minolta CR-5, Tokyo, Japan). The instrument measured the color coordinates, including L* (lightness), a* (representing the color spectrum from green (−) to red (+)), and b* (representing the color spectrum from blue (−) to yellow (+)). These measurements provided a quantitative assessment of the drink's color attributes during storage.

#### 2.5.9 Viscosity, sedimentation, cloud value, and non-enzymatic browning

Viscosity measurements of the formulations were performed using a Brookfield viscometer (Model DV2T, Middleborough, USA) with an LV-2 spindle. A 50 mL sample was placed in a standard beaker, and viscosity was recorded at a spindle speed of 100 rpm at 25 ± 1 °C. Measurements were taken for 30 s or until the reading stabilized. Results were expressed in centipoise (cP). For physical stability analysis, 10 mL of each drink sample was transferred into transparent test tubes with screw caps and stored under refrigeration. Sedimentation (%) was evaluated visually at predetermined storage intervals using a digital caliper to measure the height of the sediment layer. Sedimentation was calculated using the Eq. (4). Cloud value was determined by centrifuging 5 mL of the sample at 3500 × g for 15 min. The absorbance of the resulting supernatant was measured at 660 nm using a UV–Vis spectrophotometer, with distilled water used as the blank. Higher absorbance values indicated greater turbidity or cloudiness.

(4) $$\mathrm{Sedimentation}\;\left(\%\right)=\;\left(Hs/\;Th\right)\;\times \;100$$

*Hs* = Height of sediment

*Th* = Total height of the sample.

#### 2.5.10 Determination of pectin methylesterase (PME), polyphenol oxidase (PPO) and peroxidase (POD)

The activity of PME was determined by the method followed by Tian et al. [20]. To extract PME, the sample was homogenized with 0.05 mol/L Tris-HCl buffer solution (pH 7.5) in a 1:1 (v/v) ratio and stored at 4 °C for 12 h. The mixture was then centrifuged at 12,000 × g for 10 min at 4 °C, and the resulting supernatant was collected as the crude PME enzyme extract. For PME activity measurement, 50 mL of 1 % (w/v) pectin solution containing 0.1 mol/L NaCl was placed in a circulating water bath maintained at 30 ± 1 °C. The pH of the solution was adjusted and maintained at 7.5 using 0.03 mol/L NaOH. Once the pH was stabilized, 5 mL of the crude enzyme extract was added, and the volume of NaOH consumed over 20 min was recorded. PME activity was calculated and expressed as the percentage of pectin de-esterified over the reaction period.

Zhang et al. [21] methodology was used for the measurement of PPO and POD activity in the drink samples. For enzyme extraction, 10 mL of the sample was mixed with 10 mL of 0.05 mol/L potassium phosphate buffer (pH 7.0) containing 1 % (w/v) polyvinylpyrrolidone (PVP). The mixture was stored at 4 °C for 60 min, then centrifuged at 10,000 rpm for 15 min at 4 °C. The supernatant was collected and used as the enzyme extract for further assays. To determine PPO activity, 0.25 mL of the enzyme extract was mixed with 2.5 mL of 0.05 mol/L phosphate buffer (pH 7.0) and 0.5 mL of 0.02 mol/L catechol solution as the substrate. The increase in absorbance was recorded at 395 nm for 3 min. POD activity was assessed by mixing 0.2 mL of enzyme extract with 0.2 mL of 2 % (v/v) guaiacol and 0.05 mL of 0.5 % (v/v) hydrogen peroxide (H_2_O_2_), followed by the addition of 2.5 mL of 0.05 mol/L phosphate buffer (pH 7.0). The increase in absorbance at 470 nm was monitored over 3 min.

#### 2.5.11 Microbial analysis

The total plate count, and total yeast and mold count of all the samples were determined following the methodology outlined in the study of Mehra et al. [22]. Serial dilutions of each sample (10^−2^ to 10^−4^) were prepared using sterile 0.1 % peptone water. From each dilution, 0.1 mL was aseptically plated on respective media in triplicate. For total plate count, samples were spread on plate count agar and incubated at 37 °C for 24–48 h. For yeast and mold count, samples were spread on potato dextrose agar supplemented with 10 % tartaric acid (adjusted to pH 3.5) to inhibit bacterial growth and to favor fungal growth. The inoculated plates were incubated at 25–28 °C for 5–7 days, allowing sufficient time for both yeasts and molds to grow and differentiate. Colony-forming units (CFU) were reported as Log CFU/mL of drink samples.

### 2.6 Statistical analysis

Experiments were conducted in triplicate and results were reported as mean ± standard deviation. The experiment results were statistically analyzed using SPSS 25.0 software (SPSS Inc., Chicago, IL, USA). Two-way analysis of variance (ANOVA) with Duncan’s multiple-range test was applied to determine the significant differences between mean values at a confidence level of 95 %. Principal component analysis (PCA) was used to study the relationship and differences between functional drink samples at different preservation methods (NT, HT, UT, UOT and OT) and different storage times (0, 15, 30, 45 and 60 days) using Simca Statistical Software (Version14.1, Simca, MKS Umetrics AB).

## 3 Results and discussion

### 3.1 Characterization of UAE-NADES based *S. platensis* and orange peel extracts

The active functional ingredients derived from plant materials play a pivotal role in the formulation of functional foods due to their diverse functional composition [23]. The functional richness of *S. platensis* and orange peel extracts, as demonstrated in our study (Table 1), aligns with this perspective, highlighting their potential application in functional food development.

**Table 1:** Composition of UAE-NADES based *S. platensis* and orange peel extracts.

Parameters	Spirulina extract	Orange peel extract
Protein (mg/g)	655.40 ± 3.68	12.81 ± 4.38
Lipids (mg/g)	74.91 ± 2.63	5.58 ± 2.43
Total phenolic content (mg GAE/g)	33.46 ± 2.23	36.51 ± 2.38
Total flavonoid content (mg QE/g)	1.85 ± 0.08	28.66 ± 1.21
DPPH (%)	69.06 ± 0.86	74.04 ± 0.58
Chlorophyll *a* (mg/g)	31.76 ± 0.12	0.0
Chlorophyll *b* (mg/g)	23.78 ± 0.06	0.0
Carotenoids (mg/g)	6.93 ± 0.04	3.77 ± 0.05

*S. platensis* extracts exhibited the highest protein (655.40 ± 3.68 mg/g) and lipid content (74.91 ± 2.63 mg/g). In contrast, the protein and lipid content in orange peel extracts were significantly lower, measuring only 12.81 ± 4.38 mg/g and 5.58 ± 2.43 mg/g, respectively. However, in terms of TPC and TFC, orange peel extracts exhibited significantly higher values 36.51 ± 2.38 mg GAE/g and 28.66 ± 1.21 mg QE/g, compared to *S. platensis* extracts, which contained TPC of 33.46 ± 2.23 mg GAE/g and TFC of 1.85 ± 0.08 mg QE/g. This difference also accounts for the higher antioxidant potential of orange peel extracts (74.04 %) relative to *S. platensis* (69.06 %). The elevated values obtained through UAE-NADES further underscore the efficacy of this extraction technique in stabilizing phenolics and flavonoids while enhancing their release from cells due to extensive cellular disruption in both extracts. The *S. platensis* extracts contained chlorophyll *a* (31.76 ± 0.12 mg/g), chlorophyll *b* (23.78 ± 0.06 mg/g), and carotenoids (6.93 ± 0.04 mg/g), as determined using four different extraction methods. These findings indicate enhanced pigment stability and protection against degradation. In contrast, no chlorophyll *a* or b was detected in orange peel extracts due to the natural absence of these pigments in citrus peel. However, the carotenoid content in orange peel extracts was measured at 1.77 ± 0.05 mg/g.

Our overall composition results for *S. platensis* and orange peel extracts are higher as compared to those reported in studies, including Seghiri et al. [24], and AlFadhly et al. [25], which extensively assessed *S. platensis*. Likewise, our findings are in line with Ortiz-Sanchez et al. [26], which provided a detailed evaluation of orange peel composition. The rich nutritional profile of these extracts underscores their potential as highly effective candidates for the development of functional food products [27].

Extraction is a critical initial step, so UAE with NADES was selected due to their well-documented efficacy in extracting sensitive compounds from natural resources [28,29]. Among the various NADES evaluated, the combination of lactic acid and choline chloride was identified as the most effective, based on an extensive literature review confirming its superior extraction efficiency. US generates cavitation, where microbubbles form and collapse violently, creating localized high pressure and temperature. This mechanical effect disrupts cell walls, enhances mass transfer, and facilitates the release of compounds from algal and plant matrices. As a result, the combination of the UAE with NADES significantly enhances extraction efficiency compared to conventional methods [30,31]. This synergistic approach offers a sustainable, high-performance solution for maximizing extract yields while preserving their structural integrity and bioactivity. Key extraction parameters, including molar ratio, viscosity, temperature, pH, extraction duration, sample-to-solvent ratio, polarity, solubility, and particle size of samples, were accurately optimized to achieve maximum yield. UAE in combination with NADES demonstrated enhanced extraction efficiency, reinforcing its status as a sustainable and highly effective advanced extraction technique. Moreover, our results of high extraction efficiency using UAE-NADES are consistent with previous studies of Martins et al. [32], Viñas-Ospino et al. [33], Airouyuwa et al. [34] and Zurob et al. [35], which demonstrated the efficacy of UAE-NADES in extracting valuable substances from *S. platensis,* orange peel, date seeds and olive leaves.

### 3.2 In-vitro digestibility

The assessment of in-vitro digestibility of samples treated with different preservation methods is essential to understand their behavior under simulated gastrointestinal conditions. The highest digestibility was observed in the UOT treatment (91.20 ± 0.35 %), while the lowest was in HT (87.97 ± 0.41 %). The NT sample had a digestibility value of 89.57 ± 0.41 %, UT was 90.60 ± 0.55 %, and OT reached 89.91 ± 0.32 %. The UOT had the highest value because together, they can maximize the release of digestible nutrients while preserving bioactivity, leading to the highest digestibility among treatments. Overall, no major differences were observed among samples treated with different preservation methods, though small variations occurred. US can disrupt cell walls, enhance the release of bioactive compounds, and increase enzyme accessibility, potentially boosting digestibility. In contrast, OZ is less likely to break down complex food matrices but effectively preserves bioactive compounds and reduces microbial load, maintaining digestibility close to the control value. Heat treatment, however, may decrease in-vitro digestibility due to protein aggregation, Maillard reactions, enzyme degradation, and nutrient entrapment within heat-altered fiber networks. This study provides novel insights into the potential effects of non-thermal preservation on the digestibility of functional beverages. Cassani et al. [9] and Buniowska et al. [4] support our findings as these studies demonstrated an increase in the digestibility of strawberry drink and mango-papaya blend drink after non-thermal treatments, aligning with our results.

### 3.3 Sensory properties

**Fig. 2:**
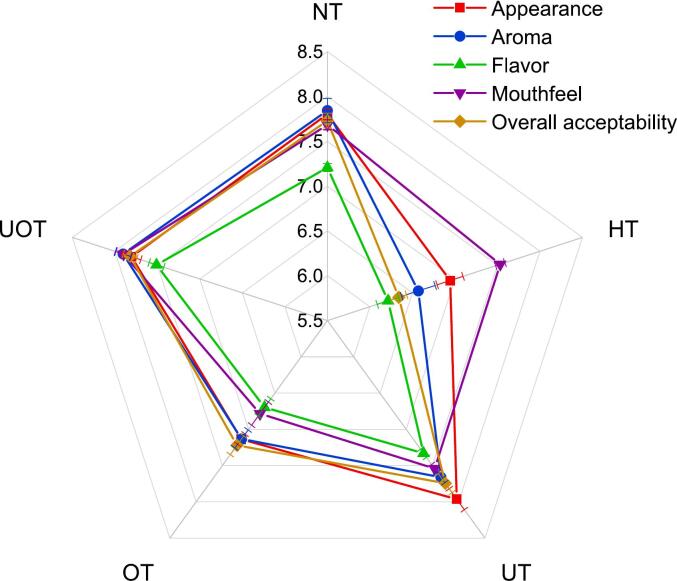
Sensory parameters (Appearance, Aroma, Flavor, Mouthfeel, Overall acceptability) of functional drink samples treated with different preservation methods (NT, HT, UT, OT, UOT).

Sensory evaluation is a critical factor in determining consumer acceptance of beverages. The strawberry-cantaloupe functional drink demonstrated high sensory appeal across all evaluated attributes, including appearance, aroma, flavor, mouthfeel, and overall acceptability. These fruits were selected for their natural sweetness, vibrant color, and distinctive aroma, which contribute to creating an exceptional functional beverage. Their combination enhances sensory properties, producing a visually striking hue that stimulates thirst and an inviting aroma that elevates the drinking experience. The blend also delivers a unique, refreshing mouthfeel, while the inherent sweetness of the fruits reduces the need for added sugars or artificial sweeteners. These qualities, as reflected in the sensory evaluation results, drive strong consumer preference and position the drink as a standout option in the functional beverage market. The results presented in Fig. 2 indicate that preservation treatments had a significant impact on the sensory properties of the samples. The highest overall acceptability score was observed in UOT (7.82), followed closely by UT (7.74), while the lowest score was recorded for HT (6.34) compared to the NT (7.73). Appearance was most preferred in UT (7.96), with NT and UOT showing similar values (7.80), while HT had the lowest appearance score (6.94). Aroma scores were highest in UOT (7.90), followed by NT (7.84), with HT scoring the lowest (6.57). Flavor scores were also highest in UOT (7.51) and lowest in HT (6.21). Similarly, the mouthfeel score was highest for UOT (7.90), while OT had the lowest score (6.68). The UOT treatment showed the highest overall acceptability and balanced sensory attributes due to the combined benefits. US enhanced appearance and mouthfeel by reducing particle size and creating a more uniform texture, while OZ prevented microbial spoilage without generating off-flavors. This combination helped maintain freshness, preserving key aroma and flavor compounds. In contrast, HT caused alteration in taste due to the production undesirable flavors, thermal degradation of pigments and volatile compounds, lowering sensory scores. The synergy of UOT effectively retained the drink’s natural qualities, leading to superior sensory outcomes. Similar findings have been reported in previous studies. For example, in the study Oladunjoye et al. [36], the authors observed a decrease in sensory scores for pasteurized hog plum juice, while US application improved sensory attributes compared to the control. Likewise, the study by Kalsi et al. [37] reported similar results, where US enhanced sensory scores of guava juice, while pasteurization led to a decline.

### 3.4 pH, titratable acidity (TA), and total soluble solids (TSS)

**Fig. 3:**
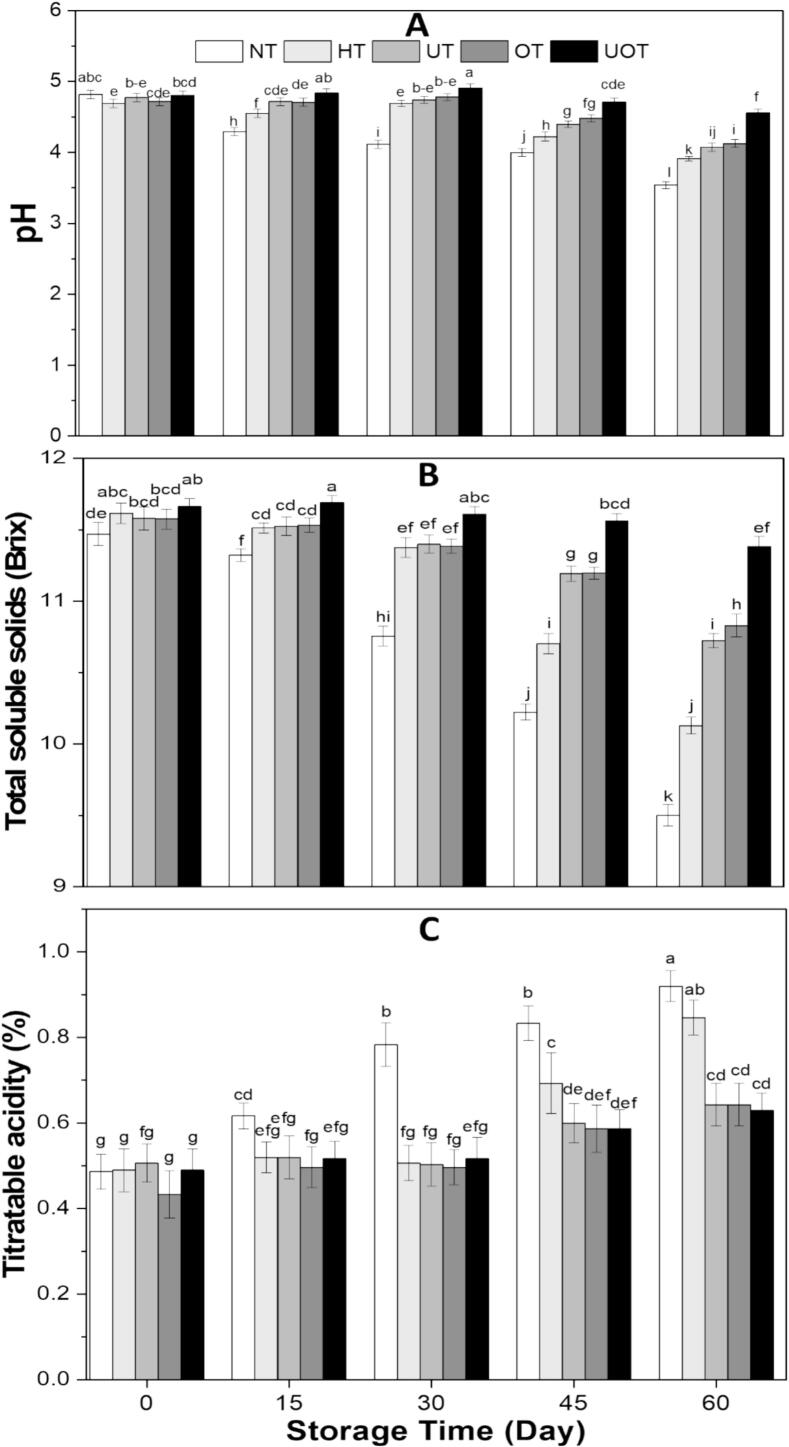
(A) pH, (B) total soluble solids, and (C) titratable acidity of functional drink samples treated with different preservation methods (NT, HT, UT, OT, UOT) at different storage times (0, 15, 30, 45, and 60 days). Treatments with different letters above bars show significant differences (P < 0.05).

pH (acidity/alkalinity), TA (titratable acidity, organic acid content), and TSS (total soluble solids, mainly sugars), are key parameters for assessing the quality and stability of beverages. The influence of various preservation methods on these parameters of samples during storage is illustrated in Fig. 3. On day 0, only slight variations were observed across treatments (HT, UT, OT, and UOT). A reduction in pH was noted in HT, potentially due to the thermal degradation of organic compounds, leading to acid release. In contrast, pH remained stable in the other treatments (Fig. 3 (A)). TSS increased across all treatments, likely because bound sugars were liberated from disrupted cell structures (Fig. 3 (B)). TA remained stable in most cases but showed a decline in OT, possibly indicating that ozone degraded certain acids or facilitated their conversion into non-acidic compounds [38] (Fig. 3 (C)). All measured parameters exhibited significant changes (p < 0.05) during storage across all treatments, with the most pronounced variations observed in the control (NT). The pH in the NT formulation declined markedly from 4.82 ± 0.06 to 4.00 ± 0.06. A similar trend was observed in HT, where pH decreased from 4.69 ± 0.06 to 3.91 ± 0.03. Notably, UOT exhibited the least reduction in pH, decreasing only slightly from 4.81 ± 0.06 to 4.56 ± 0.05, suggesting a stabilizing effect of this treatment. Conversely, TA increased as pH decreased, with the most pronounced rise observed in the control (NT), increasing from 0.49 ± 0.04 % to 0.92 ± 0.04 %. The smallest increase was recorded in the UOT sample (0.49 ± 0.05 % to 0.63 ± 0.04 %), while UT and OT exhibited similar trends. In the HT sample, TA remained stable initially but showed a rapid increase after 45 and 60 days of storage. TSS decreased from 11.47 ± 0.08°Brix to 9.50 ± 0.08 °Brix in the control (NT) and from 11.62 ± 0.07°Brix to 10.13 ± 0.06°Brix in the HT-treated sample. However, in the UOT-treated sample, TSS showed the lowest and slight reduction from 11.66 ± 0.06°Brix to 11.38 ± 0.07°Brix, highlighting the effectiveness of the combined treatment in preserving the drink's composition. It can be seen from Fig. 3 that mostly except NT all the thermally and non-thermally processed samples showed noticeable changes at 45 and 60 days of storage almost at the end. It was because with increasing storage time the microbial activity started to increase which fermented the sugars into organic acids and furthermore the oxidation and enzymes contributed to acid formation that lowered the pH and TSS and increased the acidity [39]. Our findings agreed with the findings of Herrera-Ponce et al. [40] who reported the significant decrease of pH and increase of acidity of whey-oat beverage and indicated that US treated samples demonstrated less variations in storage, Adulvitayakorn et al. [41] also provided the similar results related to pH and TA in thermal, microwave and thermosonicated sugarcane juice in storage, Rajashri et al. [42] showed the TSS reduced of all samples of tender coconut water but decrease was less in US and OZ treated samples during storage, and Fatima et al. [43] where researchers found that TSS reduced to less than half in control muskmelon and sugarcane juice but combined treatment of sonication and microwave lowered this reduction like in our study.

### 3.5 Total phenolic content (TPC), total flavonoid content (TFC), and total carotene content (TCC)

**Fig. 4:**
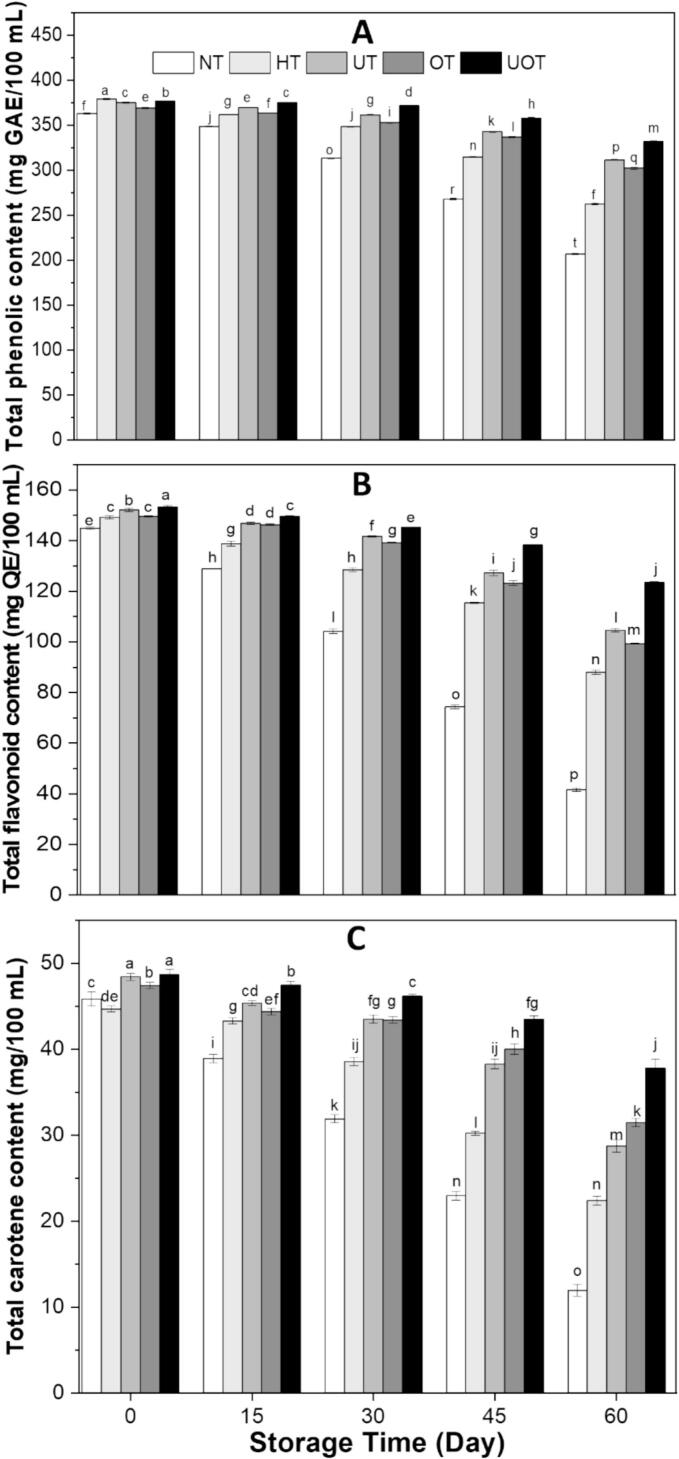
(A) Total phenolic content, (B) total flavonoid content, and (C) total carotene content of functional drink samples treated with different preservation methods (NT, HT, UT, OT, UOT) at different storage times (0, 15, 30, 45, and 60 days). Treatments with different letters above bars show significant differences (P < 0.05).

The functional drink exhibited a high concentration of bioactive compounds due to the natural richness of strawberry and cantaloupe juice. Additionally, the incorporation of the extract significantly enhanced these bioactive levels, resulting in even greater nutritional and functional benefits. Fig. 4 illustrates the variations in TPC, TFC, and TCC of the functional drink over two months of storage. These sensitive bioactive compounds exhibited a decreasing trend across all treatments during storage because of their degradation, influenced by various factors. By the end of the storage period, the lowest TPC (207.15 ± 0.86 mg GAE/100 mL), TFC (41.61 ± 0.69 mg QE/100 mL), and TCC (11.97 ± 0.72 mg/100 mL) were recorded in the control (NT), whereas as expected the highest values were observed in UOT sample with TPC (331.83 ± 0.66 mg GAE/100 mL), TFC (123.49 ± 0.44 mg QE/100 mL), and TCC (37.83 ± 0.98 mg/100 mL) indicating a synergistic protective effect of US and OZ. So, among the treatments, UOT exhibited the best retention of bioactive compounds, followed by UT, OT, and HT, respectively. US and OZ caused the enzyme inactivation, oxidation prevention, and enhanced compound extractability, reducing the loss of phenolics and flavonoids. The analogous findings have been documented by Fatima et al. [43] where authors reported that these bioactive compounds reduced in all sample especially non-treated during storage in muskmelon and sugarcane juice blend but the combined treatment of US and microwave provided the optimum retention of these sensitive compounds, Nadeem et al. [44] also provided the similar results that combined US and chemical treatment of carrot-orange juice blend provided the better retention of these bioactive compounds as compared to control and other treatments during three months of storage and Chauhan & Negi. [45] demonstrated the same findings that these compounds decreased during storage in Malay rose apple juice but US and OZ combined reduced the reduction improving the overall quality. This highlights the potential of non-thermal US and OZ, particularly in combination, as effective preservation techniques for sensitive functional drinks. Their application helps maintain bioactive compounds, which are crucial for the desired benefits of nutrient-rich functional beverages, making them the most effective non-thermal preservation methods for these beverages in the future.

### 3.6 Antioxidant activity

**Fig. 5:**
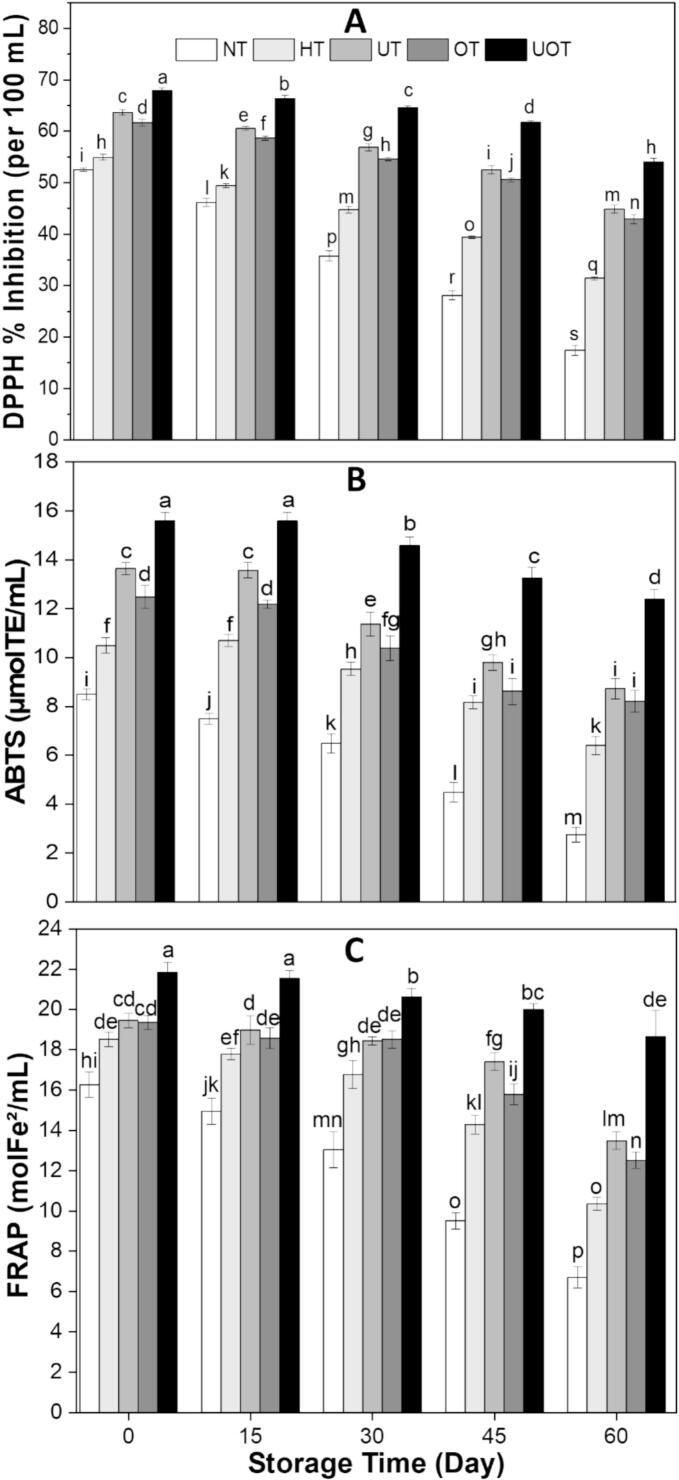
Antioxidant activity ((A) DPPH, (B) ABTS, and (C) FRAP) of functional drink samples treated with different preservation methods (NT, HT, UT, OT, UOT) at different storage times (0, 15, 30, 45, and 60 days). Treatments with different letters above bars show significant differences (P < 0.05).

Antioxidant activity refers to the ability of compounds to neutralize free radicals, preventing oxidative damage to cells and biomolecules. The antioxidant activity of the drink samples, assessed using DPPH, ABTS, and FRAP assays, is presented in Fig. 5. Following all thermal and non-thermal treatments, antioxidant activity increased across all samples. The highest enhancement was observed in UOT across all assays, followed by UT, OT, and HT, with NT showing the lowest antioxidant activity. This occurs because these treatments enhance bioactive components in fruit juices by inducing microstructural changes [46]. A declining trend in antioxidant activity was observed throughout storage, with the lowest values recorded at day 60 in NT, DPPH (17.46 ± 0.99 DPPH % Inhibition (per 100 mL)), ABTS (2.76 ± 0.30 µmolTE/mL), and FRAP (6.72 ± 0.53 molFe^2^/mL). In contrast, UOT exhibited the highest retention of antioxidant activity, with DPPH (54.07 ± 0.72 DPPH % Inhibition (per 100 mL)), ABTS (12.39 ± 0.41 µmolTE/mL), and FRAP (18.66 ± 1.30 molFe^2^/mL). It was evident that UT and OT outperformed HT in preserving antioxidant activity, as HT led to the degradation of antioxidant bioactive compounds. The combined application of both non-thermal treatments effectively safeguarded the drink’s quality. The synergistic effect of two novel technologies in better retention of antioxidant activity has also been documented by other studies. Demirok & Yıkmış. [47] reported the same results when they stored the tangerine juice treated with US and microwave together, Chauhan & Negi [45] also showed that antioxidant activity of Malay rose apple juice decreased during storage in all treatments but better retention was observed in US and OZ treated juice, similarly Herrera-Ponce et al. [40] found the decreasing trend in antioxidant activity of whey-oat beverage but US treatment slowed down the decrease and Nadeem et al. [44] also observed that combined US and chemical treatment reduced the decrease in antioxidant activity of carrot-grape juice blend during the storage.

### 3.7 Ascorbic acid

Ascorbic acid (vitamin C) is a natural antioxidant that enhances the nutritional value, stability, and oxidative protection of functional drinks. As shown in Table 2, ascorbic acid levels decreased slightly in all samples after treatment and declined significantly throughout the storage period. In HT, ascorbic acid levels decreased immediately after treatment, though the decline during storage was less severe than in NT. The smallest reduction was observed in UOT, where levels declined from 40.15 ± 0.43 mg/100 mL on day 0 to 34.51 ± 0.38 mg/100 mL on day 60. In contrast, NT exhibited the most significant decline, from 41.35 ± 0.58 mg/100 mL to 17.92 ± 0.81 mg/100 mL over the same period. Among the individual treatments, UT demonstrated better retention than OT, while the combined UOT treatment was the most effective in preserving ascorbic acid. In NT, ascorbic acid declined more during storage because the absence of preservation methods left the drink vulnerable to enzymatic oxidation and microbial activity, accelerating degradation. In contrast, treatments like US and OZ inactivated spoilage microbes and oxidative enzymes, slowing ascorbic acid breakdown over time. Our findings align with the findings of Chauhan & Negi [45] where authors proved the effectiveness of US and OZ treatment in better retention of ascorbic acid in malay rose apple juice during storage as compared to non-treated. Rajashri et al. [42] conducted research on tender coconut water, where ascorbic acid was reduced in storage more in the control as compared to the US and OZ treated samples. Basak et al. [48] also reported the highest decrease of ascorbic acid in control mixed fruit beverages as compared to pulsed light treated.

**Table 2:** Ascorbic acid, viscosity, sedimentation, cloud value, non-enzymatic browning, in-vitro digestibility and color parameters (L*, a*, b*) of functional drink samples treated with different preservation methods (NT, HT, UT, OT, UOT) at different storage times (0, 15, 30, 45, and 60 days).

Storage Time (Day)	Treatments	Ascorbic acid (mg/100 mL)	Viscosity (cP)	Sedimentation (%)	Cloud value	Non-enzymatic browning	Color values		
							L*	a*	b*
0	NT	41.35 ± 0.58^a^	9.02 ± 0.06^e^	0	0.84 ± 0.05^cde^	0.62 ± 0.06^j^	61.12 ± 0.75^ab^	21.88 ± 0.83^a^	23.59 ± 0.42^e^
	HT	37.45 ± 0.35^e^	9.53 ± 0.07^b^	1.16 ± 0.2^n^	0.74 ± 0.05^f-i^	0.61 ± 0.06^j^	58.33 ± 0.55^fg^	18.48 ± 0.51^gh^	27.01 ± 0.85^a^
	UT	39.47 ± 0.33^c^	8.16 ± 0.05^l^	0	1.02 ± 0.05^a^	0.64 ± 0.06^hij^	60.37 ± 0.41^bc^	20.86 ± 0.72^ab^	23.52 ± 0.44^e^
	OT	39.03 ± 0.47^c^	8.86 ± 0.05^fg^	0	0.84 ± 0.05^cde^	0.68 ± 0.05^g-j^	61.71 ± 0.61^a^	19.61 ± 0.38^cde^	25.35 ± 0.54^bc^
	UOT	40.15 ± 0.43^b^	8.61 ± 0.06^hi^	0	0.98 ± 0.07^ab^	0.63 ± 0.06^hij^	60.93 ± 0.36^ab^	20.62 ± 0.37^bc^	23.55 ± 0.56^e^

15	NT	36.65 ± 0.30^fg^	8.99 ± 0.07^e^	5.15 ± 0.81^j^	0.71 ± 0.07^hi^	0.72 ± 0.05^f-j^	57.44 ± 0.42^hi^	20.18 ± 0.60^bcd^	22.92 ± 0.20^ef^
	HT	37.08 ± 0.36^ef^	9.60 ± 0.05^b^	4.19 ± 0.16^k^	0.75 ± 0.04^e-i^	0.62 ± 0.06^j^	56.68 ± 0.40^ij^	18.52 ± 0.45^fgh^	25.68 ± 0.40^b^
	UT	39.15 ± 0.28^c^	8.50 ± 0.05^j^	1.05 ± 0.41^n^	0.92 ± 0.03^bc^	0.66 ± 0.04^g-j^	60.61 ± 0.36^bc^	20.87 ± 0.86^ab^	23.52 ± 0.36^e^
	OT	38.68 ± 0.35^d^	8.67 ± 0.05^h^	1.98 ± 0.29^m^	0.81 ± 0.05^def^	0.67 ± 0.05^g-j^	60.64 ± 0.33^bc^	18.81 ± 0.24^efg^	24.62 ± 0.44^cd^
	UOT	40.09 ± 0.40^b^	8.78 ± 0.05^g^	0	0.95 ± 0.05^ab^	0.63 ± 0.06^hij^	60.82 ± 0.25^b^	20.88 ± 0.58^ab^	23.88 ± 0.16^de^

30	NT	31.62 ± 0.26^j^	8.81 ± 0.07^fg^	11.38 ± 0.31^e^	0.36 ± 0.03^m^	0.81 ± 0.06^def^	54.41 ± 0.45^l^	19.55 ± 0.41^def^	21.46 ± 0.65^g^
	HT	34.55 ± 0.40^i^	9.77 ± 0.06^a^	9.13 ± 0.40^g^	0.58 ± 0.05^jk^	0.73 ± 0.06^f-i^	55.68 ± 0.40^k^	17.41 ± 0.27^i^	25.10 ± 0.41^bc^
	UT	38.75 ± 0.67^d^	8.81 ± 0.06^fg^	3.22 ± 0.28^l^	0.81 ± 0.04^def^	0.70 ± 0.05^g-j^	59.28 ± 0.60^de^	20.15 ± 0.59^bcd^	23.31 ± 0.96^e^
	OT	37.55 ± 0.32^e^	8.49 ± 0.06^j^	4.60 ± 0.34^k^	0.77 ± 0.05^e-h^	0.70 ± 0.04^g-j^	58.84 ± 0.27^ef^	18.47 ± 0.53^gh^	23.53 ± 0.37^e^
	UOT	39.45 ± 0.42^c^	8.89 ± 0.03^f^	1.08 ± 0.28^n^	0.90 ± 0.03^bcd^	0.65 ± 0.04^h-j^	59.82 ± 0.25^cd^	20.98 ± 0.79^ab^	23.55 ± 0.48^e^

45	NT	25.31 ± 0.56^m^	8.56 ± 0.05^ij^	16.71 ± 0.57^c^	0.34 ± 0.06^m^	0.86 ± 0.05^cd^	49.34 ± 0.34^n^	15.70 ± 0.43^j^	19.82 ± 1.10^h^
	HT	31.48 ± 0.38^j^	9.40 ± 0.07^c^	13.53 ± 1.15^d^	0.51 ± 0.07^k^	0.76 ± 0.05^efg^	52.08 ± 0.81^m^	17.27 ± 0.46^i^	23.63 ± 0.44^e^
	UT	36.29 ± 0.35^gh^	9.17 ± 0.05^d^	6.52 ± 0.34^i^	0.71 ± 0.09^hi^	0.72 ± 0.06^f-j^	57.68 ± 0.45^gh^	19.55 ± 0.47^def^	23.18 ± 1.03^e^
	OT	35.71 ± 0.34^h^	8.26 ± 0.05^k^	8.04 ± 0.33^h^	0.67 ± 0.04^ij^	0.74 ± 0.05^fgh^	57.08 ± 0.42^hij^	18.50 ± 0.51^fgh^	22.99 ± 0.24^ef^
	UOT	38.58 ± 0.47^d^	9.07 ± 0.05^e^	2.12 ± 0.43^m^	0.82 ± 0.05^def^	0.72 ± 0.03^f-j^	58.49 ± 0.43^efg^	20.10 ± 0.43^bcd^	23.43 ± 0.38^e^

60	NT	17.92 ± 0.81^o^	8.03 ± 0.07^m^	21.57 ± 1.00^a^	0.24 ± 0.04^n^	1.20 ± 0.08^a^	45.51 ± 0.45^o^	12.70 ± 0.89^k^	16.56 ± 0.47^i^
	HT	24.45 ± 0.42^n^	9.25 ± 0.07^d^	18.34 ± 0.28^b^	0.39 ± 0.04^lm^	0.97 ± 0.08^b^	48.71 ± 0.49^n^	16.64 ± 0.46^i^	21.96 ± 0.14^fg^
	UT	29.45 ± 0.43^k^	8.84 ± 0.07^fg^	10.28 ± 0.82^f^	0.53 ± 0.06^k^	0.86 ± 0.06^cde^	53.78 ± 0.55^l^	18.45 ± 0.38^gh^	22.92 ± 0.21^ef^
	OT	27.66 ± 0.31^l^	8.12 ± 0.05^lm^	13.24 ± 0.78^d^	0.42 ± 0.07^l^	0.95 ± 0.04^bc^	52.38 ± 0.53^m^	17.50 ± 0.44^hi^	22.92 ± 0.19^ef^
	UOT	34.51 ± 0.38^i^	9.00 ± 0.03^e^	5.80 ± 0.91^j^	0.72 ± 0.05^ghi^	0.76 ± 0.05^efg^	56.46 ± 0.43^j^	18.76 ± 0.87^efg^	22.83 ± 0.86^ef^

Data are expressed as means ± SD. Values with different letters in each column are significantly different (P < 0.05).

### 3.8 Color parameters

Color is a key sensory attribute in drinks that influences consumer perception, indicates ingredient composition, and reflects product quality and stability. The variations in color values (L*, a*, b*) of the drink samples are presented in Table 2. The observed color changes in the drink samples during storage can be attributed to pigment degradation, oxidation reactions, enzymatic activity, and Maillard reactions, which vary depending on the applied treatment [36,48]. In HT-treated samples, L* and a* values decreased while b* values increased immediately after treatment, whereas non-thermal treatments preserved the initial color values. However, color changes occurred during storage in the following order: NT > HT > OT > UT > UOT. In NT, the values shifted from L* (61.12 ± 0.75), a* (21.88 ± 0.83), and b* (23.59 ± 0.42) at 0 days to L* (45.51 ± 0.45), a* (12.70 ± 0.89), and b* (16.56 ± 0.47) at 60 days. In contrast, UOT effectively retained color stability, with L* (60.93 ± 0.36), a* (20.62 ± 0.37), and b* (23.55 ± 0.56) at 0 days, which only slightly changed to L* (56.46 ± 0.43), a* (18.76 ± 0.87), and b* (22.83 ± 0.86) after 60 days, highlighting its superior potential in maintaining the drink’s color compared to other thermal and non-thermal treatments. Similarly in research by de Albuquerque et al. [38] the color of the nopal beverage changed during storage but the US and other non-thermal treatments prevented the significant degradation of color as occurred in control sample. Basak et al. [48] also demonstrated a similar trend in their study about decreasing (L*, a*, b*) values in control mixed fruit beverages as compared to somewhat maintained values in pulsed light treated sample.

### 3.9 Viscosity, sedimentation, cloud value, and non-enzymatic browning

Viscosity determines the drink's texture and mouthfeel, sedimentation indicates the degree of particle settling over time, cloud value reflects the stability of suspended particles affecting visual appeal, and non-enzymatic browning results from Maillard reactions and caramelization, impacting color and flavor. The variations in viscosity, cloud values, and non-enzymatic browning over two months of storage across all treatments are presented in Table 2. These parameters were influenced by both thermal and non-thermal treatments, leading to noticeable differences at 0 days, followed by further changes throughout storage. In NT, viscosity showed a decreasing trend from 9.02 ± 0.06 cP to 8.03 ± 0.07 cP. In HT, viscosity initially increased (Heat-induced gelatinization of polysaccharides and denaturation of proteins), reaching a peak of 9.77 ± 0.06 cP at 30 days before declining to 9.25 ± 0.07 cP. Non-thermal treatments caused a significant initial decrease in viscosity at 0 days, with the lowest observed in UT (8.16 ± 0.05 cP), but viscosity increased until day 45 in all treatments except OT, where it continuously declined, reaching 8.12 ± 0.05 cP. In UOT, viscosity decreased to 8.61 ± 0.06 cP at 0 days but gradually increased to 9.00 ± 0.03 cP by day 60. These results indicate complex variations in viscosity across different treatments. The significant decrease in viscosity observed in UT, OT and UOT could be attributed to molecular breakdown and cell wall disruption, leading to reduced structural integrity of hydrocolloids Oliveira et al. [49]. However, the subsequent increase in viscosity (except for OT) may be due to the re-association of polysaccharides and proteins, forming a more stable matrix over time. In OT-treated samples, the continuous decline in viscosity suggests potential oxidative degradation of hydrocolloids and the breakdown of stabilizing agents due to ozone exposure. Siddique et al. [50] also reported similar results related to changes in the viscosity of heat-treated and US-treated kinnow-whey-based beverage during 90 days of storage. Sedimentation increased progressively across all treatments throughout the storage period, starting from 0 % on day 0, except for HT, which had an initial value of 1.16 ± 0.2 %. Heat denatures proteins and pectin, causing them to lose solubility and form larger aggregates. The initial small sedimentation (1.16 %) right after heating is due to immediate protein coagulation. By day 60, the highest sedimentation was observed in NT (21.57 ± 1.00 %), while the lowest was recorded in UOT (5.80 ± 0.91 %). UT showed a sedimentation value of 10.28 ± 0.82 %, followed by OT (13.24 ± 0.78 %) and HT (18.34 ± 0.28 %). The rise in sedimentation was attributed to particle aggregation, phase separation, and the degradation of stabilizing compounds over time. UOT, UT and OT effectively minimized sedimentation by enhancing particle dispersion, reducing particle size, and improving colloidal stability, whereas NT and HT facilitated settling due to weaker stabilization and thermal degradation. Cloud value was lowest in HT (0.74 ± 0.05) immediately after treatment and highest in UT (1.02 ± 0.05) at day 0. During storage, a decreasing trend was observed across all samples, with the lowest final value in NT (0.24 ± 0.04) and the highest, most stable value in UOT (0.72 ± 0.05) observed at 60 days. The decline in cloud value in HT was attributed to thermal-induced protein denaturation, pectin degradation, and particle aggregation, resulting in reduced turbidity. UOT effectively preserved cloud stability by enhancing particle dispersion and colloidal interactions, preventing excessive clarification [49]. Vollmer et al. [51] also obtained similar findings that not treated and thermally treated pineapple juice samples showed low cloud value and more sedimentation as compared to non-thermally treated sample in 14 days of storage. There were no significant differences in non-enzymatic browning among all samples at 0 days, but the values increased during storage [36]. The highest increase was observed in NT, rising from 0.62 ± 0.06 to 1.20 ± 0.08, while the lowest increase was recorded in UOT, from 0.63 ± 0.06 to just 0.76 ± 0.05. UOT effectively minimized browning by reducing oxidative stress and preserving antioxidants, thereby slowing down color deterioration. In comprehensive study Zhu et al. [52] demonstrated the effectiveness of non-thermal technologies in preventing non-enzymatic browning of juices as compared to thermal methods.

### 3.10 Pectin methylesterase (PME), polyphenol oxidase (PPO) and peroxidase (POD) activity

**Fig. 6:**
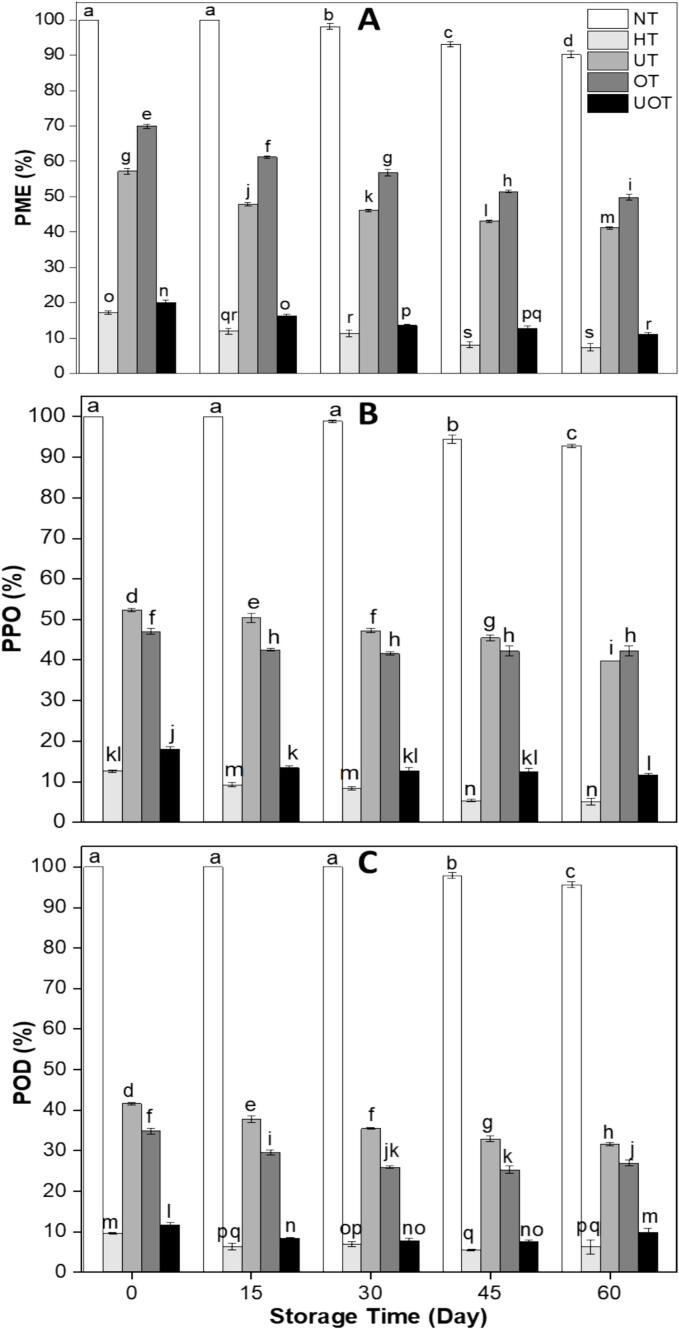
(A) Pectin methylesterase (PME), (B) polyphenol oxidase (PPO), and (C) peroxidase (POD) activity of functional drink samples treated with different preservation methods (NT, HT, UT, OT, UOT) at different storage times (0, 15, 30, 45, and 60 days). Treatments with different letters above bars show significant differences (P < 0.05).

Enzymes in drinks influence quality by affecting color, clarity, texture, and stability, with their activity varying based on processing and storage conditions. The variations in PME, PPO, and POD activity are illustrated in Fig. 6. Thermal and non-thermal treatments significantly reduced enzyme activity, while in NT, enzyme activity remained at 100 %. HT and UOT were the most effective in reducing enzyme activities at 0 days compared to UT and OT. PME activity decreased to 17 % and 20 %, PPO to 13 % and 18 %, and POD to 10 % and 12 % in HT and UOT, respectively, significantly lower than UT and OT. Moreover, enzymatic activities further declined throughout storage in all samples including NT. Thermal treatments (HT) were most effective in reducing PME, PPO, and POD activity due to protein denaturation and enzyme inactivation at high temperatures and synergistic oxidative stress. In UOT, enzyme inactivation occurred due to US-induced mechanical disruption (cavitation, shear forces, and localized high pressure) and OZ-induced oxidative damage (modification of amino acid residues and structural disruption). This combined non-thermal approach effectively reduced PME, PPO, and POD activities without the need for heat. The results of our research totally align with Kalsi et al. [37], who showed that enzymatic activity was at peak in non-treated guava juice but it decreased to its lowest values by heat treatment. US also decreased the enzymatic activities but was lower than the heat treatment. Moreover, Vollmer et al. [51] also reported similar findings related to the inactivation of PME, PPO and POD in pineapple juice treated thermally and non-thermally. In another research conducted by Panigrahi et al. [53] the analogous findings have been documented related to enzymes inactivation in sugarcane juice by combined treatment of ultrafiltration and OZ.

### 3.11 Microbial properties

**Fig. 7:**
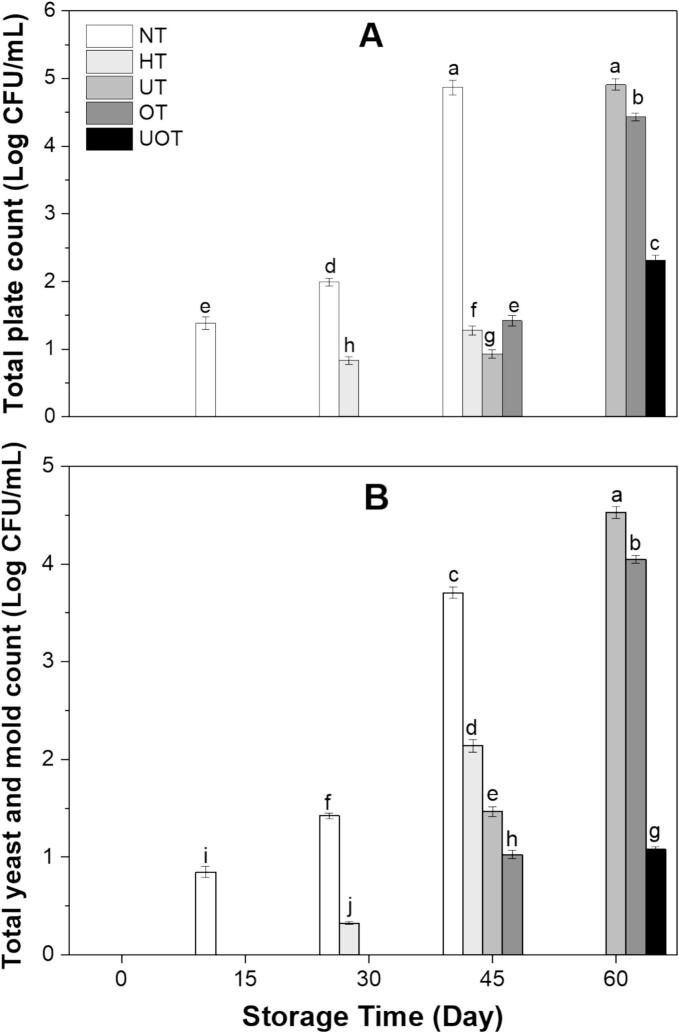
Effect of the different preservation methods (NT, HT, UT, OT, UOT) on (A) total plate count, and (B) total yeast and mold count of functional drink samples at different storage times (0, 15, 30, 45, and 60 days). Treatments with different letters above bars show significant differences (P < 0.05).

Microbes in drinks including bacteria, yeasts, and molds, can influence quality, bioactivity, safety and shelf life by causing spoilage. The total plate count and total yeast and mold count of drink samples during different storage days are shown in Fig. 7. On day 0, no microbial presence was observed in any sample because of the presence of (5 + 5 %) concentration of algal and waste extracts. Many studies have been conducted on the use of natural extracts as preservatives in beverages, demonstrating exceptional results in their ability to enhance preservation [55]. These natural extracts have shown significant antimicrobial, antioxidant, and shelf-life-extending properties, making them a promising alternative to synthetic preservatives [10]. Microbial counts in the NT sample continued to increase steadily throughout the storage period, reaching uncountable levels exceeding 5 Log CFU/mL by day 60. In the HT samples, microbial growth became evident at day 45 and similarly reached uncountable levels by day 60. In contrast, the treated samples exhibited varying degrees of microbial control. On day 60, the UT samples showed a total plate count of 4.91 ± 0.09 Log CFU/mL and a total yeast and mold count of 4.52 ± 0.09 Log CFU/mL. The OT samples had a total plate count of 4.43 ± 0.06 Log CFU/mL and a total yeast and mold count of 4.04 ± 0.04 Log CFU/mL. Notably, the UOT demonstrated the most effective microbial inhibition, with a total plate count of 2.32 ± 0.07 Log CFU/mL and a total yeast and mold count of 1.07 ± 0.03 Log CFU/mL. HT was initially effective in eliminating microbes, but over time, surviving spores or recontamination led to significant microbial growth, particularly after 45 days. In contrast, UOT maintained the lowest microbial counts even at 60 days, likely due to its persistent antimicrobial effects through structural modifications in microbial cells, oxidative stress, and interactions with bioactive compounds. While microbial growth in UOT treated samples may continue under favorable storage conditions, the increase would be significantly slower compared to NT and HT. This study showed that this novel functional drink processed with combined non-thermal processing UOT can be stored safely for up to 60 days. There are plethora of studies showing the similar results related to reduction of microbes by non-thermal processing and slow growth during storage such by Panigrahi et al. [53] who applied combined treatment of ultrafiltration and OZ on sugarcane juice, Mehta et al. [54] who applied US, ultraviolet and cold plasma on tomato based beverage, Oladunjoye et al. [36] conducted research on application of US on hog plum fruit juice, de Albuquerque et al. [38] worked on US treatment of nopal beverage, and Rajashri et al. [42] where authors treated tender coconut water with US and OZ.

### 3.12 Principal component analysis (PCA)

**Fig. 8:**
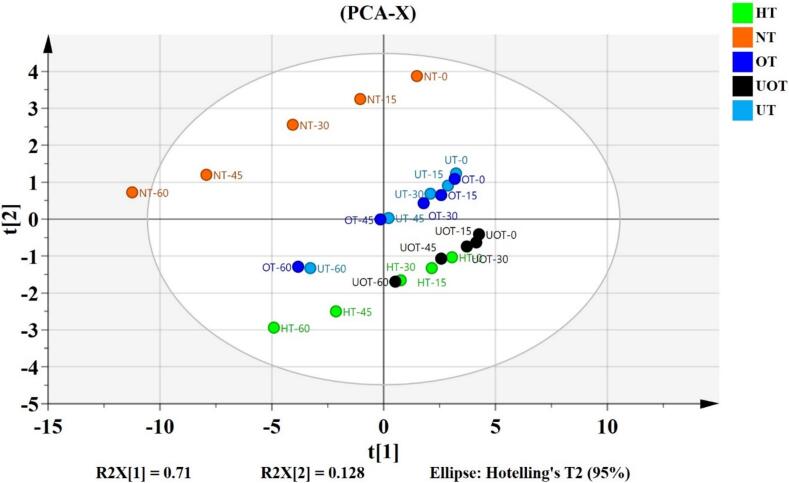
Principal component analysis (PCA) of functional drink samples treated with different preservation methods (NT, HT, UT, OT, UOT) at different storage times (0, 15, 30, 45 and 60 days).

PCA was used to determine the effect of the preservation methods (NT, HT, UT, OT, UOT) on overall quality parameters of a novel functional drink of strawberry and cantaloupe juice blend incorporated with *S. platensis* and orange peel extracts at different storage times (0, 15, 30, 45 and 60 days) as illustrated in Fig. 8. The PCA score plot reveals the distribution, with PC1 and PC2 explaining 83.8 % of the total variation. The first component, PC1, was responsible for 71 % of the total variation, while the second component (PC2) was responsible for 12.8 %. A distinct separation of the different preservation methods of functional drink samples (HT, UT, OT, UOT) from the control (NT) indicates that the preservation methods significantly influence the overall characteristics of the novel functional drink of strawberry and cantaloupe juice blend incorporated with *S. platensis* and orange peel extracts. Samples separated by PC1 are more different than samples separated by PC2. Additionally, samples with similar overall profiles are clustered together, such as UT and OT samples.

## 4 Conclusion

In this research, a novel clean-label functional drink was developed using strawberry and cantaloupe juice blends, added with clean-label extracts of *S. platensis* and orange peel, and preserved through thermal and non-thermal treatments. The findings revealed that the synergistic effect of US and OZ was the most effective preservation method during two months of storage, outperforming individual US and OZ treatments. Thermal pasteurization, on the other hand, was less effective, as it degraded the drink's quality parameters due to the heat sensitive nature of this novel drink. Untreated sample spoiled by day 45 due to elevated enzymatic and microbial activity, which compromised overall quality. The combined US-OZ treatment (UOT) successfully maintained physicochemical parameters, preserved bioactivity, reduced enzymatic and microbial activity, improved digestibility, enhanced sensory attributes, and significantly extended the drink’s shelf life. This research marks an important step forward in developing and preserving health-promoting beverages using natural ingredients and innovative sustainable preservation technologies, eliminating the need for synthetic preservatives or thermal treatments. The study highlights the potential of carefully optimized combined non-thermal technologies to enhance shelf life while retaining the nutritional value of sensitive beverages a critical area of ongoing research. This research also paves the way for long-term preservation of clean-label functional beverages through the application of combined non-thermal technologies, complemented by aseptic Tetra Pak packaging, which is widely used in the commercial market. Additionally, the research findings suggest that these functional drinks, when treated with non-thermal technologies, could sustain a shelf life of 2–4 months, especially given their natural antimicrobial and antioxidant properties. However, further research is essential to explore the individual and combined effects of other emerging non-thermal technologies on similar beverages. Scaling up these methods for industrial application is crucial to assess their feasibility for commercial production, promoting sustainability and eco-friendliness. Market analysis is also recommended to evaluate consumer acceptance and preferences for functional beverages preserved through non-thermal techniques, paving the way for broader adoption of clean-label, health-focused products in the commercial market.

## Research involving human participants and/or animals

Sensory analysis involving human participants was conducted following ethical guidelines. Informed consent was obtained from all participants prior to the evaluation.

## CRediT authorship contribution statement

**Kashmala Chaudhary:** Writing – original draft, Visualization, Methodology, Formal analysis, Data curation, Conceptualization. **Samran Khalid:** Writing – review & editing, Writing – original draft, Methodology, Formal analysis, Data curation, Conceptualization. **Najla AlMasoud:** Writing – review & editing. **Taghrid S. Alomar:** Writing – review & editing. **Sadia Ansar:** Writing – review & editing. **Ahmed Fathy Ghazal:** Writing – review & editing, Formal analysis, Visualization. **Abderrahmane Aït-Kaddour:** Writing – review & editing, Funding acquisition. **Rana Muhammad Aadil:** Writing – review & editing, Supervision, Methodology, Formal analysis, Data curation, Conceptualization.

## Declaration of competing interest

The authors declare that they have no known competing financial interests or personal relationships that could have appeared to influence the work reported in this paper.

## Data Availability

Data will be made available on request.
